# Preoperative Fasting Time and Its Association with Hypoglycemia during Anesthesia in Pediatric Patients Undergoing Elective Procedures at Tikur Anbessa Specialized Hospital, Addis Ababa, Ethiopia

**DOI:** 10.1155/2021/9166603

**Published:** 2021-07-14

**Authors:** Hussien Endris Assen, Anissa Mohammed Hassen, Ananya Abate, Bikis Liyew

**Affiliations:** ^1^Department of Anesthesia, College of Medicine and Health Science, University of Gondar, Gondar, Ethiopia; ^2^School of Public Health, College of Medicine and Health Science, Wollo University, Dessie, Ethiopia; ^3^Department of Anesthesiology, College of Medicine and Health Science, Addis Ababa University, Addis Ababa, Ethiopia; ^4^Department of Emergency and Critical Care Nursing, School of Nursing, College of Medicine and Health Sciences, University of Gondar, Gondar, Ethiopia

## Abstract

**Background:**

Preoperative fasting is important to reduce the risk of pulmonary aspiration during anesthesia. The influence of prolonged fasting time on glucose levels during anesthesia in children remains uncertain. Therefore, this study is aimed at assessing preoperative fasting time and its association with hypoglycemia during anesthesia in pediatric patients undergoing elective procedures at Tikur Anbessa Specialized Hospital, Addis Ababa, Ethiopia. The research hypothesis of the study is as follows: there is a prolonged preoperative fasting time, and it influences the glucose levels during anesthesia among pediatric patients undergoing elective procedures at Tikur Anbessa Specialized Hospital, Addis Ababa, Ethiopia.

**Methods:**

Institutional based cross-sectional study was conducted among 258 pediatric patients who had undergone elective procedures in a tertiary care center. A systematic sampling method was used to select study participants. The data were collected through face-to-face interviews and medical record reviews. Binary logistic regression was used to identify associated factors of hypoglycemia during anesthesia among pediatric patients undergoing elective procedures. All explanatory variables with a *p* value of ≤0.25 from the bivariable logistic regression model were fitted into the multivariable logistic regression model to control the possible effect of confounders, and finally, the variables which had an independent association with hypoglycemia were identified based on adjusted odds ratio with 95% confidence interval, and a *p* value less than 0.05 was significant.

**Results:**

The mean (standard deviation) fasting hours from breast milk, solid foods, and clear fluids were 7.75 (2.89), 13.25 (3.14), and 12.31 (3.22), respectively. The majority (89.9%, 57.9%, and 100%) of participants had fasted from solid, breast milk, and clear fluids for more than 8, 6, and 4 hours, respectively. More than one-fourth (26.2%) of participants were hypoglycemic immediately after induction. Residence, order of nothing per mouth, source of patient, and duration of fasting from solid foods had a significant association with hypoglycemia during anesthesia in children.

**Conclusion:**

Children undergoing elective procedures were exposed to unnecessarily long fasting times which were associated with hypoglycemia during anesthesia.

## 1. Introduction

American Society of Anesthesiologists defined preoperative fasting as a prescribed period before a procedure when patients are not allowed the oral intake of fluid or solids [1–4]. Children are required to fast before anesthesia to reduce the volume of the stomach content to reduce the risk of regurgitation and aspiration of gastric contents during the procedure [5]. Different guidelines recommend the minimum fasting period of 2 hours for clear liquids and 6 hours for solids [1, 3, 6–12]. The European Society of Anesthesiologists also suggested a more liberal preoperative fasting protocol for clear liquids (one hour before anesthesia) [13]. Despite the progressive development of guidelines, patients continue to undergo unnecessarily prolonged preoperative starvation to mitigate the risk of aspiration [6]. However, children who were denied oral fluids for more than 6 hours preoperatively did not benefit in terms of intraoperative gastric volume and pH as compared with children who were permitted unlimited fluids up to 2 h preoperatively [14].

Prolonged fasting leads to dehydration, biochemical imbalance, and hypoglycemia, especially in children, and has been discouraged in anesthetic and surgical practice [15–17]. Perioperative hypoglycemia would lead to patient morbidity and mortality, and it is a danger in pediatric practice resulting in anesthetic problems including, lethargy, irritability, metabolic acidosis, and seizures [18]. Prolonged fasting times have several negative implications and lead to poor anesthetic outcomes [19, 20]. Normally, there is a rise in the plasma glucose level in response to surgical stress in normal adults, but children do not respond with a hyperglycemic reaction to the same degree [21]. In Ethiopia, most of the patients fasted from food and fluid longer than the time recommended by the international guidelines. In our setup, the inability to measure glucose when deemed necessary due to inconsistent availability of glucometer makes glucose management more difficult during anesthesia. This is further complicated by a lack of data regarding the incidence of hypoglycemia during anesthesia with a tendency to give dextrose without blood glucose level measurement. The absence of clearly adopted national and institutional preoperative fasting protocols and poor implementation of international guidelines due to lack of knowledge and compliance among health care professionals makes it difficult to predict preoperative fasting times in our patients [3]. Therefore, this study was aimed at assessing the duration of preoperative fasting times and its association with hypoglycemia during anesthesia among pediatrics patients. The statistical hypothesis of the study is as follows: HO (null hypothesis) = preoperative normal fasting times among pediatric patients undergoing elective procedures at Tikur Anbessa Specialized Hospital, Addis Ababa, Ethiopia, and *H*_A (alternative hypothesis)_ = preoperative long fasting times among pediatric patients undergoing elective procedures at Tikur Anbessa Specialized Hospital, Addis Ababa, Ethiopia.

## 2. Methods

### 2.1. Study Setting

The study was conducted at Tikur Anbessa Specialized Hospital which is found in Addis Ababa (the capital city of Ethiopia) in the Lideta subcity. According to the central statistical agency of Ethiopia (CSA), as of 2013, the town of Addis Ababa has a total population of 3,130,673, of which 1,478,890 are men and 1,624,783 women. It is the nation's largest and highest referral hospital. This hospital sees approximately 370,000–400,000 patients a year, but the exact number is not known. It has 700 beds. The hospital is the largest tertiary care teaching center in the country where many subspecialty services are delivered including pediatric surgery. The hospital is planned and accommodated and facilitated with the outpatient department (OPD), which has seven X-ray, nine surgical, and two diagnostic laboratory rooms; internal medicine, gynecological and obstetrics, surgical, pediatrics, and emergency departments; and referral clinics (chest, renal, neurology, cardiology, dermatology, sexually transmitted diseases, gastrointestinal, infectious diseases, orthopedics, general surgical, gynecologic and obstetrics, diabetic, hematology, and medical intensive care units).

### 2.2. Study Design and Period

An institution-based cross-sectional study was employed from June to October 2019, at Tikur Anbessa Specialized Hospital (TASH) which is located in Addis Ababa, the capital city of Ethiopia.

### 2.3. Study Population

All pediatric patients aged less than 14 years old who have been scheduled for elective procedures were the study populations. All pediatric patients aged 0-14 years, who had undergone elective procedures (elective surgery, MRI, gastrointestinal endoscopy, bronchoscopy, bone marrow aspiration, and biopsy), were included. And patients with the known metabolic or endocrine disorders were excluded from the study.

### 2.4. Sample Size

The required sample size was determined by the single population proportion formula, considering the proportion of hypoglycemia among pediatric patients as computed using the formula with the input 95% confidence level (*z*) and 5% margin of error (*d*) and estimating a proportion (*p*) 50%,n = [(Z*α*/2)2 × P (1 − *P*)]/*d*2 = 384.2 ≈ 384. However, the number of pediatric patients who were scheduled for elective procedures and sedation for the past three consecutive months was 600. So we have used a correction formula and the final sample size was 258.

### 2.5. Sampling Technique

A systematic sampling method was used to select study participants. All unique medical registration numbers of a child who came for elective procedures from June to October 2019 were selected from the elective surgery registration logbook and were sorted based on their unique medical registration number in an ascending order (1, 2, 3, 4, etc.) By using a systematic sampling technique, children's charts were selected until the required sample size is obtained in every 2^nd^ (where 2 = *N*/*n* 600/258). The first study subject was the first one when it was selected by lottery method from the first 2 of children. Then, the selected children were contacted on the day of their schedule, and the data collectors extracted the required data from the selected charts and through face-to-face interviews.

### 2.6. Data Collection Tools and Procedures

Information about study variables was collected by three BSc nurses through face-to-face interviews of the caregivers and review of patient's medical records using chart extraction form and filled to a structured questionnaire adapted from different works of literature. The questionnaire incorporated sociodemographic data of the patients and the caregivers, clinical data, and questions related to preoperative fasting durations of the patients as reported by the caregivers. A preoperative fasting (NPO) time is the duration between the time patients last took any form of an enteral meal including clear fluids and breast milk and the time of induction. Information about preoperative fasting times was collected through face-to-face interviews with the caregivers. The capillary blood glucose level at the fingertip was measured using a capillary glucometer (ON-Call Extra) immediately after induction of anesthesia (but before the procedure begins), at the end of the procedure, and at a random time for those procedures whose procedure time took more than one hour. Any glucose level less than 54 mg/dl was reported as hypoglycemia [22], and glucose level > 200 mg/dl was considered as hyperglycemic [23], and we made sure that the first glucose measurement was done before the patient was given any form of IV dextrose. Sociodemographic information like age and sex of patients and caregivers as well as educational status, marital status, and the job of the caretakers was collected through face-to-face interviews with the caregivers. Other clinical variables like type of surgery and American Society of Anesthesiologists (ASA) class were collected through reviewing medical charts of the patients. Children were categorized as ASA class I if they were well built and had no limitation during walking or playing; ASA class II if they had advanced malignancy (example hematologic malignancy) and if they were grossly malnourished or had mild limitation of walking, playing, or other age-appropriate activities; and ASA class III if they had active heart or lung problems, if they were debilitated or bedridden, or if they were premature infants with postconception age less than 60 weeks.

### 2.7. Data Quality Management

Before collecting our data, the data extraction checklist was prepared in English and pretested to gather relevant data from the medical records. To ascertain the data quality, data collection was conducted by three BSc nurses. The training was given to the data collectors on how to fill the questionnaire, clarification of the whole study tools, variables, and research ethics. Continuous monitoring and supervision were done by the principal investigator every day for completeness and clarification of the data. Pretest was conducted on 5% of the sample size at TASH, and necessary corrections and modifications were done on the study tool.

### 2.8. Data Processing and Analysis

Data clean-up and cross-checking were done before analysis. Data was checked; recoded and completed questionnaires were given identification numbers and entered to SPSS version 25 for analysis. Binary logistic regression was used to identify associated factors of hypoglycemia just after induction among pediatric patients undergoing elective procedures at Tikur Anbessa Specialized Hospital. Variables with a *p* value of ≤0.25 from the bivariable logistic regression model were fitted into the multivariable logistic regression model to control the possible effect of confounders, and finally, the variables which had an independent association with were identified based on AOR with 95% CI, and a *p* value less than 0.05 was significant. Data were entered, cleaned, and coded into SPSS, V25 software, and for analysis. Simple descriptive statistics such as frequency mean and standard deviations were calculated. Then, to see the relationship of independent variables with the dependent variable, binary logistic regression was performed, and to see the effect of each independent variable on the dependent variable, multivariable logistic regression was calculated. As a result, crude and adjusted odds ratio with a 95% confidence interval was calculated. A *p* value of less than or equal to 0.05 was considered significant.

## 3. Result

### 3.1. Sociodemographic Characteristics of Study Participants

There were 258 eligible respondents, and none of them refused to participate giving a response rate of 100%. The mean age of participants was (mean ± SD) 4.9 ± 3.8 of which 43% are within the group of 5-14 years and the majority 171 (66.3%) of them were male. Most of the caregivers (69) (26.7%) had completed primary education, and 45 (17.4%) were unable to read and write; eighty-eight (34.1%) of the caregivers were merchants while 45 (17.4%) were housewives/jobless. Most of the caregivers (246) (95.3%) were married. Similarly, a considerable proportion of 184 (71.3%) came for the service outside of Addis Ababa (Table 1).

### 3.2. Clinical Characteristics of Study Participants

Among the study participants, 168 (65.1%) were outpatient, while the rest 90(34.9%) were inpatient. All patients were given instructions about preoperative fasting. One hundred thirty-two (51.2%) participants were told to make their child NPO after midnight, while 105 (40.7%) before midnight. The majority (235) (91.1%) of the patients were ASA class II. Among the 7 (2.7%) patients who had comorbidities, 4 (57.1%) had a neurologic problem and 3 (42.9%) had a renal problem. One hundred twenty-seven (49.2%) were under general anesthesia, and 128 (49.6%) use the intravenous induction technique. The minimum, maximum, and mean (± SD) duration of procedure and anesthesia was 15, 270, 59 ± 23, and 20, 290, 77 ± 30 minutes, respectively. Among the study participants, 57 were on breastfeeding, 207 took solid foods, and 218 took clear liquids before the procedure. The minimum, maximum, and mean (SD) fasting hours from breast milk were 3, 13, and 7.75 (2.89), in which the majority 33 (57.9%) have fasted longer than 6 hours. The minimum, maximum, and mean (SD) fasting hours from solid foods were 5, 20, and 13.25 (3.14). The majority (95.2%) of patients have fasted from solid foods longer than 6 hours. Similarly, the minimum, maximum, and mean (SD) fasting hours from clear liquids were 5, 20, and 12.31 (3.22) (Table 2).

### 3.3. Glucose Measurement during Anesthesia

The minimum, maximum, and mean glucose level of patients immediately after induction is 30, 199, and 91.16 mg/dl with a standard deviation of 39.68. Sixty-two (26.2%) patients had a blood glucose level less than 54 mg/dl and were considered hypoglycemic, while 73.8% had more than 54 mg/dl. Similarly, minimum, maximum, and mean glucose level of patients at the end of surgery is 34, 290, and 134.18 mg/dl with a standard deviation of 71.15. Among the 150 patients who did not take intraoperative glucose, 8 (5.3%) patients became hypoglycemic at the end of surgery and all of them were induced with intravenous anesthetics and had fasted from solid food for more than 8 hours preoperatively, while none of the patients who took intraoperative glucose were found to be hypoglycemic at the end of surgery. On the contrary, among 108 patients who took intraoperative glucose, 14 (7.71%) of them became hyperglycemic at the end of surgery (glucose level > 200 mg/dl).

### 3.4. Factors Associated with Hypoglycemia during Anesthesia

Bivariate and multivariable logistic regression analysis was performed, and those variables with *p* < 0.25 in the bivariate association were included in the model. Thus, age of the patient, educational status of caregiver, marital status of caregiver, residence, order of instruction given to the caregivers to make their child NPO, ASA class, induction technique, time of induction, source of the ward, duration of fasting from solid foods, and duration of fasting from breast milk were significantly associated with hypoglycemia during anesthesia. In the multivariable analysis, association of age of the patient, educational status, ASA class, marital status of caregiver, induction technique, induction time, and duration of fasting from breast milk with hypoglycemia turned nonsignificant, while residence, order of NPO, source of patient, and duration of fasting from solid foods had a significant association with hypoglycemia during anesthesia. Those patients who came to seek treatment from outside of Addis Ababa were 2.3 times more likely to be hypoglycemic than those who came from Addis Ababa. In comparison with those patients who fasted before midnight; those patients who fasted after morning were 95% less likely to be hypoglycemic during anesthesia (OR = 0.05, *p* < 0.006). Similarly, those who were inpatient were 70% more likely to be hypoglycemic than outpatients (OR = 1.7, *p* < 0.03). Besides, those patients who fasted more than 8 hrs were 2.3 times more likely to be hypoglycemic during anesthesia than those who fasted less than 6 hrs (OR = 2.3, *p* < 0.002) (Table 3).

## 4. Discussion

The preoperative fasting duration of pediatric patients in this study was beyond the standard recommendations. Similar findings were observed in many other studies done in different countries [18]. Reasons contributing to prolonged fasting in our setup could be the lack of adopted local protocols that promote shorter preop fasting adhering to standard guidelines. There is also a trend of ordering NPO overnight or after midnight among clinicians involved in the perioperative care of children, including anesthesia providers, irrespective of the time of going to operation theatre/schedule time, and there is no practice of revising the operation lists and ordering patients to take food and/or fluid whenever surgery was delayed. Besides, the majority of the patients were residents outside of Addis Ababa and outpatients contributing to prolonged fasting. This may be due to the tendency of clinicians to order these patients to be NPO overnight who lack close supervision. Although the consensus cut-off point for the definition of intraoperative hypoglycemia is not set forth up to our knowledge, the varying prevalence was reported ranging from 10% to 29% [18] using different cut-off points (40 mg/dl, 50 mg/dl, and 60 mg/dl). In our study, a glucose level of 54 mg/dl or less was reported as hypoglycemia according to the 2017 diabetic care consensus joint statement [22]. The prevalence of hypoglycemia (26.2%) in this study was found to be higher than studies conducted in London, Liverpool, Nigeria, and Denmark [18]. This discrepancy can be explained by the fact that the hypoglycemia threshold used by those studies was 40 mg/dl as compared to this study which is 54 mg/dl. Besides, most of the outpatient patients are not administered preoperative maintenance fluids in our setup; therefore, this might contribute to the high prevalence of hypoglycemia during anesthesia. In this study, the mean glucose level of patients after induction of anesthesia was 91.16 mg/dl (SD = 39.68). This is higher than studies conducted in other studies [24–26]. The possible reason might be due to the effect of increased glucocorticoids and stress, a defense mechanism to meet glucose requirement of tissues, saving energy, and improving intravascular volume through increasing osmolarity. In this study, the mean glucose level of patients at the end of surgery is 134.18 mg/dl (SD = 71.15). This is higher than a study conducted in Iran in which the mean blood glucose level after surgery was 101.17 (SD = 92) [27]. In this study, none of the patients who took intraoperative glucose were found to be hypoglycemic at the end of surgery. This is consistent with a study conducted in India [25]. Duration of fasting from solid foods was found to be an important factor for hypoglycemia during anesthesia which is in line with one of the Indian studies [21], but this effect can be ameliorated by administration of glucose intraoperative. Although allowing clear fluids up to 2 hours before surgery was safe and said to improve patient comfort and wellbeing, only 25.2% of caregivers reported any sign of thirst, hunger, or anxiety despite all of them fasting for more than 6 hours, and duration of fasting from clear fluids or breast milk was not associated with hypoglycemia in this study. Most studies, however, conducted in Nigeria, Liverpool, and London have reported no association between preoperative fasting duration and intraoperative hypoglycemia [18]. The time of the last meal may influence glucose homeostasis irrespective of the duration of fasting as those patients who fasted after morning were less likely to be hypoglycemic despite prolonged fasting. This is in contrast to the idea that night-time fasting may be well tolerated than daytime fast as was suggested by Nancy [28]. This could be due to the relative increment of peripheral insulin resistance in the afternoon and lower insulin level after glucose administration in the afternoon than in the morning [29]. The finding that inpatients were more likely to be hypoglycemic may be related to the invasiveness of the procedure as children's glycemic response is not proportional to the stressfulness of the procedure, unlike adults [30]. Besides, inpatients may be sicker than outpatients with depleted fat and glycogen stores. It was also suggested that outpatients may be less susceptible to hypoglycemia [31].

The reason why children from outside of Addis Ababa are more likely to develop hypoglycemia during anesthesia is unclear, but it could be related to the level of malnutrition as it is more common in children outside of Addis Ababa [32]. Besides, those patients who came outside of Addis have fasted for a prolonged time beyond the recommendation.

## 5. Limitation of the Study

Although it is the first of our country which may help as a baseline for further studies and a relatively larger sample size than most previous studies in the literature, our study is not without limitations. The inability to compare outcomes and intraoperative complications and failure to control possible confounders including the level of nutritional status with anthropometric measurements, the effect of anesthesia drugs used, preinduction bolus fluid, type, and amount of last meal were the limitations of the study.

## 6. Conclusion and Recommendation

Children undergoing elective procedures were exposed to unnecessarily long fasting times as compared to standard guidelines which are associated with hypoglycemia during anesthesia. Duration of fasting from solid foods, source of patient, and residence were important factors associated with intraoperative hypoglycemia. Therefore, hospital administrators should facilitate and coordinate with clinicians for the development of local fasting protocols that comply with standard guidelines and reinforcement mechanisms of adherence for clinicians involved in the perioperative care of children are required. Besides, anesthesia providers should consider monitoring blood glucose levels during operations or use of intraoperative glucose, especially for those who fast from solid food for more than 8 hours and those who are inpatients. And we recommend further research with a strong design and large sample size to be implemented.

## Figures and Tables

**Table 1:** Sociodemographic characteristics of pediatric patients undergoing elective procedures at Tikur Anbessa Specialized Hospital, Addis Ababa, 2019.

Variables	Category	Frequency (*N*)	Percent (%)
Age of the patients in years	<1	48	18.6
	1-4	99	38.4
	5-14	111	43.0

Sex of the patient	Male	171	66.3
	Female	87	33.7

Relation of the caregivers to the patient	Mother	162	62.8
	Father	93	36.0
	Brother	3	1.2

Age of the caregivers in years	<35	142	55
	36-50	98	38
	51-65	18	7

Educational status of the caregivers	Unable to read and write	45	17.4
	Able to read and write	38	14.7
	Primary education	69	26.7
	Secondary education	64	24.8
	College and above	42	16.3

Occupation	Housewife/jobless	75	29.1
	Government employee	37	14.3
	Nongovernment employee	34	13.2
	Farmer	24	9.3
	Merchant	88	34.1

Marital status of the caregivers	Married	232	89.9
	Single	12	4.7
	Divorced	14	5.4

Residence	Addis Ababa	74	28.7
	Outside of Addis Ababa	184	71.3

**Table 2:** Clinical characteristics of pediatric patients undergoing elective procedures at Tikur Anbessa Specialized Hospital, Addis Ababa, Ethiopia, 2019.

Variables	Category	Frequency (*N*)	Percent (%)
Told to make NPO	Yes	258	100

Who told you to make your child NPO	Surgical team	99	38.4
	Anesthesia team	60	23.3
	Pediatrician	89	34.5
	Nurses	10	3.9

Order of NPO	Before midnight	105	40.7
	After midnight	132	51.2
	After morning	21	8.2

Have you noticed signs of thirst, hunger, or anxiety	Yes	65	25.2
	No	193	74.8

ASA classification	ASA class I	8	3.1
	ASA class II	235	91.1
	ASA class III	15	5.8

Comorbidities	Yes	7	2.7
	No	251	97.3

Source	Inpatient	90	34.9
	Outpatient	168	65.1

Induction time	Morning	177	68.6
	Afternoon	81	31.4

Duration of procedure (minute)	≥45	152	58.9
	<45	106	41.1

Duration of fasting from solid foods (in hours)	≤6	10	4.8
	6-8	11	5.3
	>8	186	89.9

Duration of fasting from clear fluids (in hours)	>4	218	100

Duration of fasting from breast milk (in hours)	≤4	5	8.8
	4-6	19	33.3
	>6	33	57.9

Type of anesthesia	General anesthesia	127	49.2
	Sedation	131	50.8

Induction technique	Inhalational	50	19.4
	Intravenous	128	49.6
	Combined IV and inhalation	80	31.0

Intraoperative glucose administered	Yes	108	41.9
	No	150	58.1

NPO: nothing per mouth; IV: intravenous line; ASA: American Society Of Anesthesiologists.

**Table 3:** Multivariable logistic regression of sociodemographic and clinical variables with hypoglycemia just after induction among pediatric patients undergoing elective procedures at Tikur Anbessa Specialized Hospital, Addis Ababa, Ethiopia, 2019.

Variables	Category	Hypoglycemic (≤54 mg/dl)	Normoglycemic (>54 mg/dl)	COR (95% CI)	AOR (95% CI)	*p* value
Residence	Addis Ababa	10 (18.5)	64 (31.4)	1	1	
	Outside of Addis Ababa	44 (81.5)	140 (68.6)	2.01 (0.95-4.25)	2.3 (1.02-5.35)	0.001^∗^

Order of NPO	Before midnight	13 (24.1)	92 (45.1)	1	1	
	After midnight	36 (66.7)	96 (47.1)	0.37 (0.18-0.75)	0.22 (0.33-4.45)	0.61
	After morning	5 (9.3)	7 (3.4)	0.19 (0.05-0.72)	0.06 (0.009-0.45)	0.006^∗^

Source	Outpatient	37 (68.5)	131 (64.2)	1	1	
	Inpatient	17 (31.5)	73 (35.8)	1.21 (0.63-2.30)	1.71 (1.01-4.97)	0.03^∗^

Duration of fasting from solid (in hours)	≤6	4 (9.3)	6 (3.7)	1	1	
	6-8	4 (9.3)	7 (4.3)	1.16 (0.2-6.8)	0.3 (0.02-6.43)	0.43
	>8	35 (81.4)	151 (92.1)	2.87 (0.77-10.7)	2.3 (1.45-19.35)	0.002^∗^

^∗^Significant at *p* < 0.05. COR: crude odds ratio; AOR: adjusted odd ratio; NPO: nothing per mouth; CI: confidence interval.

## Data Availability

The data sets used and/or analyzed during the current study are available from the corresponding author on reasonable request.

## Abbreviations

AAU: Addis Ababa University
ASA: American Society of Anesthesiology
DS: Dextrose saline
ENT: Ear, nose, throat
ESA: European Society of Anesthesiologist
GA: General anesthesia
IM: Intramuscular
IV: Intravenous
LR: Ringer lactate
NPO: Nil per os
SD: Standard deviation
SPSS: Statistical Package for Social Science
TASH: Tikur Anbessa Specialized Hospital
UK: United Kingdom
WU: World Health Organization.
